# Drugs and Medicines

**Published:** 1887-04

**Authors:** 


### DRUGS AND MEDICINES.

### AND THE EVIL OF "SUBSTITUTION."

Some forty years ago, Congress passed a very stringent law prohibiting the importation of adulterated drugs, and ordering such to be sent out of the country, or destroyed. After having passed the Custom House, the purest drugs are liable to all sorts of adulteration at the hands of druggists and compounders of nostrums, and we regret to say, that this evil has increased of late years to an alarming extent.

It is claimed that many druggists are in the habit of *substituting* less costly preparations of their own in filling prescriptions, a practice not alone dishonest, but dangerous to health to a degree that calls for State legislation and penalties severe enough to put an end to the nefarious business.

We are not very much in favor of drugs, at least of everlasting dosing for every real or imaginary ailment, but oftentimes they are absolutely necessary as remedial agents ; on such occasions it becomes important, alike to physician and patient, that the remedy called for should be identical with the filled prescription.

Some forty years ago, two Cincinnati druggists got at loggerheads over a patent nostrum, sold by both under a flaming label, as "Compound Liverwort and Tar," but it came out upon the trial that neither litigant used a particle of either liverwort or tar in the preparation, and so the matter ended in a drawn game between two confessed frauds.

There are numberless cases of just such frauds now. *Put 'em out, say we!*

